# Metformin, SGLT2 Inhibitors, and Intermittent Fasting: Convergent Pathways to Healthspan Extension, Cardiovascular Longevity, and Cancer Prevention—A Narrative Review

**DOI:** 10.3390/cimb48101008

**Published:** 2026-09-30

**Authors:** Dragos Cozma, Cristina Tudoran, Cristina Vacarescu

**Affiliations:** 1 Institute of Cardiovascular Diseases Timisoara, 300310 Timisoara, Romania; dragos.cozma@umft.ro (D.C.); cristina.vacarescu@umft.ro (C.V.); 2 Cardiology Department, “Victor Babeș” University of Medicine and Pharmacy Timisoara, 300041 Timisoara, Romania; 3 Research Center of the Institute of Cardiovascular Diseases Timisoara, 300310 Timisoara, Romania; 4 Department VII, Internal Medicine II, Discipline of Cardiology, “Victor Babeș” University of Medicine and Pharmacy Timisoara, E. Murgu Square, Nr. 2, 300041 Timisoara, Romania; 5 County Emergency Hospital “Pius Brinzeu”, L. Rebreanu, Nr. 156, 300723 Timisoara, Romania; 6 Center of Molecular Research in Nephrology and Vascular Disease, “Victor Babeș” University of Medicine and Pharmacy, E. Murgu Square, Nr. 2, 300041 Timisoara, Romania

**Keywords:** metformin, SGLT2i, intermittent fasting, healthspan, cardiovascular longevity, autophagy, cancer chemoprevention, gut microbiome, geroprotection, heart failure

## Abstract

The biology of aging and the epidemiology of cardiovascular disease share a common molecular pathway expressed through nutrient-sensing kinases, mitochondrial redox state, and chronic inflammation. Three interventions have independently emerged as translators of that interconnection into clinical benefit: metformin, a biguanide that has evolved from a first-line anti-diabetic into the world’s most extensively studied putative geroprotector; the sodium–glucose cotransporter-2 inhibitors (SGLT2is), pharmacological generators of a fasting-mimetic state that has reshaped heart failure outcomes across the ejection-fraction spectrum; and intermittent fasting (IF), the evolutionary archetype from which both agents partially derive their effects on longevity. Based on the synthetized evidence, we propose a stage-stratified clinical framework for integrated deployment. To find the manuscripts relevant for our narrative review, we searched the medical literature by using key words (“metformin”; “SGLT2i”; “intermittent fasting”; “health span”; “cardiovascular longevity”; “cancer chemoprevention”; “gut microbiome”; “geroprotection”; “heart failure”) combined through Boolean operators (AND/OR). This narrative review integrates preclinical mechanistic evidence, landmark randomized controlled trial data, and emerging data on cancer chemoprevention and gut microbiome remodeling to provide a rigorous, side-by-side analysis of all three strategies. We delineate molecular convergence on adenosine monophosphate kinase (AMPK) activation, mechanistic target of rapamycin complex 1 (mTORC1) suppression, enhanced autophagy, mitochondrial renewal, inflammatory responses, and anti-cancer biology, while mapping critical divergences in specific populations, lifespan timing, combinability, and safety. Our proposed framework has to be treated with caution, as it is based on the current evidence base, which lacks dedicated outcome trials for IF and published randomized clinical trial data for metformin in non-diabetic adults.

## 1. The Convergence of Longevity Biology and Cardiovascular Medicine

Cardiovascular (CV) disease (CVD) remains the leading cause of death in industrialized nations, yet some of the biological mechanisms underlying both atherosclerotic cardiovascular disease (ATCVD) and heart failure (HF)—like chronic nutrient-sensing dysregulation, mitochondrial dysfunction, sterile inflammation, and cellular senescence—are in principle modifiable by interventions already in clinical or dietary use.

The concept of healthspan (the portion of life spent free from major morbidities) has emerged as a clinically actionable target complementary to longevity per se. CV health and healthspan are closely interrelated: the heart is simultaneously a barometer of systemic metabolic aging and a determinant of functional independence. Interventions that address the hallmarks of aging may therefore carry cardioprotective and, as this review explores, potentially oncoprotective effects, although the strength of evidence varies substantially across domains.

**Figure 1:**
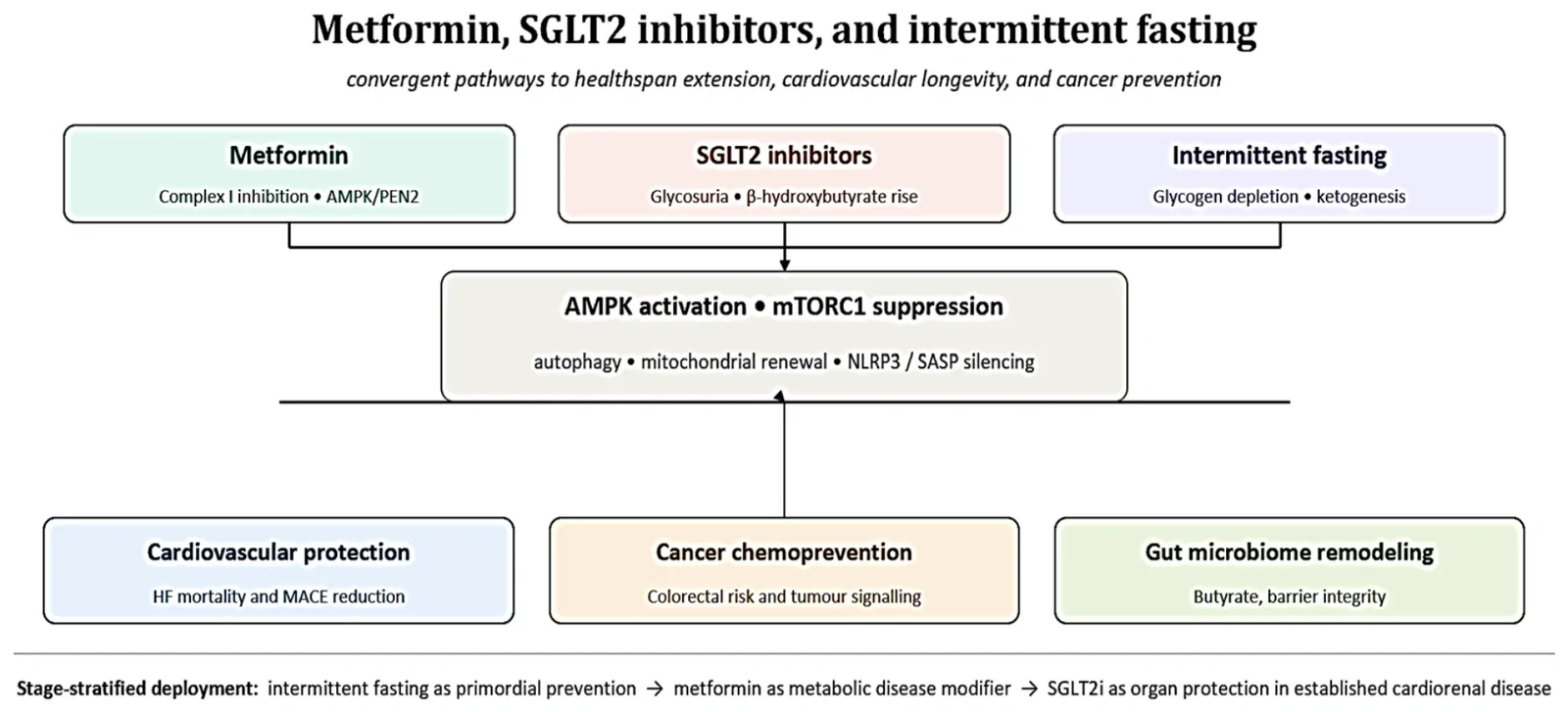
Common pathways between metformin, SGLT2is, and IF and their main effects.

Metformin has accumulated mechanistic and epidemiological evidence suggesting age-attenuating and cancer-preventive properties extending beyond glycemic control. These effects are now formally evaluated in the TAME trial (Targeting Aging with Metformin) [1,2]. Sodium–glucose cotransporter-2 inhibitors (SGLT2is) have become a cornerstone of HF therapy with consistent mortality benefit across the ejection-fraction spectrum, accompanied by emerging oncological benefits [3,4,5,6,7] (see Figure 1). Intermittent fasting (IF) is the evolutionary archetype whose downstream metabolic signature both agents partially replicate, and whose microbiome-remodeling effects overlap with, yet remain mechanistically distinct from, those of the pharmacological interventions [8,9].

Although glucagon-like peptide-1 (GLP-1) receptor agonists (GLP-1RA) have some similar effects to IF, we decided to exclude this category of drugs from our review due to several reasons:

- Semaglutide and tirzepatide produce substantial cardiometabolic benefit and are intentionally excluded from this comparative analysis, for reasons of conceptual focus rather than value judgement.
- GLP-1RA-induced weight loss is associated with 25–39% of total lost mass being lean tissue [10], as suggested by data published in several clinical trials, but the degree of lean-mass attrition and its possible long-term musculoskeletal and immunological consequences are not yet resolved by available data—another reason why we did not include GLP-1RA in this review.
- Unlike metformin, SGLT2is, or IF, GLP-1RAs do not reliably induce ketogenesis or autophagy at clinically used doses, placing them outside the convergent adenosine monophosphate kinase (AMPK)–rapamycin complex 1 (mTORC1)–autophagy framework examined here.
- Long-term implications of progressive lean-mass attrition for healthspan warrant a dedicated comparative analysis, suitable for a future review.

This narrative review pursues five objectives: (1) delineate molecular mechanisms linking each strategy to hallmarks of aging; (2) compare CV outcome evidence with critical appraisal of trial populations and limitations; (3) present the emerging oncological and microbiome evidence, with explicit acknowledgement of its preliminary nature; (4) address synergy, dangerous combinations, and lifespan timing; and (5) propose a stage-stratified clinical framework based on current evidence.

## 2. Mechanisms of Action: Three Routes to Convergent Molecular Targets

Metformin, SGLT2is, and IF converge on an overlapping set of intracellular signaling nodes that are master regulators of biological aging. The central axis is the AMPK–mTORC1–autophagy cascade, flanked by insulin/Insulin-like Growth Factor (IGF-1) signaling, sirtuin–nicotinamide adenine dinucleotide (NAD)+ metabolism, NLRP3 inflammasome activity, and cellular senescence pathways. The mechanistic evidence reviewed here derives predominantly from preclinical models and mechanistic human studies; causal attribution in clinical populations requires adequately powered prospective trials.

### 2.1. Metformin: The Lysosomal Energy Sensor and Its Downstream Cascade

Metformin’s primary molecular target is mitochondrial respiratory-chain complex I, whose partial inhibition raises the intracellular adenosine monophosphate (AMP): adenosine triphosphate (ATP) ratio and triggers AMPK via the canonical liver kinase B1 (LKB1) pathway [11]. At low, clinically relevant concentrations, a parallel lysosomal mechanism operates: metformin binds presenilin enhancer 2 (PEN2), a component of the v-ATPase–regulator–AXIN complex, activating AMPK via ATPase H^+^-transporting accessory protein 1 (ATP6AP1) independently of energetic stress [12]. This mechanistic duality partially explains efficacy at doses well below those required for maximal complex I inhibition [13].

AMPK activation orchestrates a coherent geroprotective program: mTORC1 suppression via tuberous sclerosis complex 1 (hamartin) and 2 (tuberin) (TSC1/2) phosphorylation and Rag guanosine triphosphate (GTP)-mediated inhibition; Unc-51-like autophagy-activating kinase 1 (ULK1) dephosphorylation with consequent autophagy induction; elevation of NAD+ activating SIRT1–FOXO3a–PGC-1α; and nuclear factor kappa-light-chain-enhancer of activated B cells (NF-κB) suppression reducing NLRP3 inflammasome assembly and the senescence-associated secretory phenotype (SASP) [13,14,15,16,17]. Mechanistically, metformin engages several of the hallmarks of aging described by Kulkarni et al., although the extent to which these preclinical observations translate to the human aging process at therapeutic doses remains under investigation [1].

Metformin does not cause lactic acidosis through AMPK activation at therapeutic doses in hemodynamically stable patients with adequate renal clearance. Lactic acidosis occurs when metformin accumulates due to impaired renal elimination (estimated glomerular filtration rate (eGFR) < 30 mL/min/1.73 m^2^) combined with tissue hypoxia (sepsis, myocardial infarction, severe hepatic failure), which drives anaerobic glycolysis independently of metformin’s mechanism. The risk is pharmacokinetic (drug accumulation) rather than mechanistic; this distinction is clinically important when counselling patients about the drug’s safety profile [18].

Two independent randomized clinical trials published in *Aging Cell* (2019) demonstrated that metformin co-administration during structured exercise training significantly attenuates the mitochondrial biogenic response to both resistance [19] and aerobic [20] exercise in older adults, blunting maximal oxygen consumption (VO_2_max) improvements and muscle hypertrophic responses. For metabolically healthy, physically active individuals, metformin may thus partially antagonize the healthspan benefits of physical training—a finding with direct implications for its off-label use in non-diabetic subjects. These results have not yet been replicated at scale; however, prescribers should discuss this trade-off with exercise-active patients.

### 2.2. SGLT2 Inhibitors: Pharmacological Architects of a Sustained Fasting-Mimetic State

SGLT2is block proximal tubular glucose reabsorption, inducing glycosuria of approximately 88–90 g/day (~200–280 kcal), creating a sustained caloric deficit without appetite suppression [21]. The consequent fall in insulin and rise in the glucagon–insulin ratio shifts hepatic metabolism towards fatty-acid β-oxidation and ketogenesis, producing circulating β-hydroxybutyrate (β-OHB) concentrations of approximately 0.3–0.5 mM, metabolically analogous to early fasting [22].

This fasting-mimetic state activates AMPK and suppresses mTORC1 in cardiac, renal, and hepatic tissues, with evidence of restored autophagic flux in preclinical models of hyperglycemia and obesity [22,23]. In the cardiac microvascular endothelium, empagliflozin activates AMPK, phosphorylates dynamin-related protein 1 (DRP1) (inhibiting pathological mitochondrial fission), and promotes mitofusin 1 and 2 (MFN1/2)-mediated fusion, normalizing the mitochondrial architecture. These findings are derived from in vitro and animal studies and await confirmation in human cardiac tissue [24]. β-OHB additionally inhibits NLRP3 inflammasome assembly via direct protein binding [25] and suppresses class I histone deacetylases (HDACs), reprogramming gene expression in patterns convergent with caloric restriction. Katsuumi et al. demonstrated canagliflozin-mediated elimination of senescent cells with downstream alleviation of pathological aging phenotypes in mice—mechanistically compelling data that require human translational validation [26].

SGLT2is can precipitate ketoacidosis in patients without diabetes mellitus, particularly those with HF and reduced pancreatic reserve. Combining SGLT2is with prolonged fasting can favor the onset of euglycemic ketoacidosis (euDKA). SGLT2is lower blood glucose by causing glycosuria, which reduces circulating glucose availability, lowers endogenous insulin secretion, and increases glucagon (direct α-cell effects and via hypoglycemia signaling). Prolonged fasting (typically >12–14 h) also depletes glycogen and shifts metabolism from glucose to fatty-acid use and increases lipolysis and hepatic ketone production. These combined effects drive a lower insulin-to-glucagon ratio, promote lipolysis and hepatic ketogenesis, and reduce renal clearance of ketone bodies (SGLT2is increase reabsorption of acetoacetate) [27]. Because plasma glucose may be only mildly elevated, the diagnosis is frequently delayed. Key precipitants include prolonged fasting, perioperative nil-by-mouth periods, intercurrent illness with vomiting, very-low-carbohydrate diets, and concurrent IF regimens. SGLT2is should be withheld 3–4 days before elective surgery and are contraindicated alongside prolonged therapeutic fasting (>24 h). All prescribers and patients must be familiar with sick-day rules [28].

### 2.3. Intermittent Fasting: The Evolutionary Archetype

IF encompasses time-restricted eating (TRE) (6–10-h feeding windows), alternate-day fasting (ADF), and the 5:2 protocol, all producing recurrent transitions between fed and fasted metabolic states [8,9]. After approximately 12–14 h of abstinence, hepatic glycogen is substantially depleted, insulin falls, glucagon rises, and β-hydroxybutyrate (β-OHB) reaches 1–3 mM, higher than SGLT2i-induced levels, engaging AMPK–mTORC1–autophagy signaling [8,25].

The re-feeding phase is not merely anabolic recovery but an active regenerative event: mTORC1 reactivation drives protein synthesis and mitochondrial biogenesis, incorporating components renewed during the preceding catabolic phase. This feast–famine cycling represents a biological distinction from both chronic caloric restriction and the continuous fasting-mimicry of SGLT2is [9,29].

Epidemiological observations from religious fasting practices (Ramadan consistently improving insulin sensitivity, blood pressure, body weight, and inflammatory markers in both healthy and metabolically at-risk populations [30]) provide supporting evidence, although confounders including caloric restriction, sleep pattern changes, and social behaviors preclude causal inference.

### 2.4. Convergence and Synergy: Mechanistic Evidence and Clinical Caution

Table 1 compares mechanistic effects across six aging-relevant pathways. All three interventions achieve AMPK activation, mTORC1 suppression, enhanced autophagy, reduced reactive oxygen species (ROS) emission, NLRP3 silencing, and alleviation of cellular senescence in preclinical models. The mechanistic evidence suggests potentially additive effects when combining IF with metformin (distinct upstream AMPK inputs) or IF with SGLT2is on autophagy and anti-inflammatory endpoints. However, it must be emphasized that these synergistic effects are mechanistic inferences from preclinical data; no adequately structured clinical trial has evaluated combined efficacy or safety.

SGLT2is chronically elevate β-OHB to 0.3–0.5 mM; prolonged fasting independently raises β-OHB to 1–3 mM. Their combination creates a risk of additive ketogenesis and euglycemic diabetic ketoacidosis (DKA) even without pre-existing diabetes. Short-duration TRE (16:8) alongside SGLT2is is likely manageable in non-insulin-dependent, well-educated patients who are monitored for symptoms. Extended fasts (>24 h), very-low-carbohydrate diets, perioperative fasting, vomiting, or any febrile illness will require temporary SGLT2i discontinuation (hold 3–4 days before and resume after tolerating normal oral intake) [27].

All three interventions converge on the same core geroprotective molecular network in preclinical models. Evidence strength for human translation varies by pathway and is discussed in the text.

## 3. Cardiovascular Outcomes: The Clinical Evidence Hierarchy

### 3.1. Metformin: Durable Cardioprotection in the Insulin-Resistant Phenotype

Metformin’s CV evidence originates with the United Kingdom Prospective Diabetes Study (UKPDS) overweight subgroup (1998), where newly diagnosed type 2 diabetes mellitus (T2DM) patients randomized to metformin demonstrated a 39% reduction in myocardial infarction (MI) and a 36% reduction in all-cause mortality versus conventional treatment at 10-year follow-up [36]—benefits that persisted a further decade post-trial (the “legacy effect”), suggesting durable vascular biology modification rather than mere glycemic adjustment. A systematic review of 40 studies (>1 million patients with coronary artery disease) found metformin to be associated with 33% lower all-cause mortality (adjusted HR 0.67) and 19% lower CV mortality (HR 0.81), with the greatest benefit in patients with concurrent HF or recent MI^35^ see Table 2. An umbrella review confirmed approximately 20% lower major CV event risk in T2DM [37].

The Targeting Aging with Metformin trial (TAME) is enrolling 3000 non-diabetic adults aged 65–79 with ≥1 age-related condition to evaluate metformin (1500 mg/day) versus placebo on a composite of incident T2DM, CV events, cancer, dementia, and all-cause mortality—the first FDA-regulated clinical trial positioning biological aging itself as the therapeutic target [2,38]. No survival benefit in non-diabetic patients has been demonstrated by this RCT to date; this reflects absence of evidence rather than evidence of absence, pending TAME completion.

Metformin is safe in stable HF [39] and should be withheld at eGFR < 30 mL/min/1.73 m^2^ and during acute illness causing hemodynamic compromise [18].

### 3.2. SGLT2 Inhibitors: A Paradigm Shift in HF Pharmacotherapy

The EMPA-REG OUTCOME trial (2015) delivered the first genuine CV mortality reduction with a glucose-lowering agent in the modern era: 38% relative reduction in CV death and 32% in all-cause mortality with empagliflozin in T2DM patients with established CVD [3]. The benefit profile—early onset (within 3 months), concentrated on HF and sudden cardiac death rather than MI or stroke, and disproportionate to modest glycemic effects—strongly implicates hemodynamic and metabolic mechanisms rather than anti-atherosclerotic ones. While these data represent a landmark advance, it should be noted that the EMPA-REG population was highly selected (established CVD, mean age 63 years, 74% male), and that benefit magnitude may differ in primary prevention populations.

The CANVAS program (canagliflozin) [40] and DECLARE-TIMI 58 (dapagliflozin) [41] confirmed the class effect on HF hospitalization reduction; CANVAS showed a higher incidence of lower-limb amputation (hazard ratio (HR) = 1.97), contextualized as concentrated in patients with pre-existing peripheral artery disease or foot ulcers. Dedicated HF trials (DAPA-HF [5] and EMPEROR-Reduced [6]) each demonstrated approximately 25% reduction in the composite of CV death and worsening HF in HF with reduced ejection fraction (HFrEF), predominantly driven by a ~30% reduction in hospitalization. The pooled meta-analysis of five major SGLT2i HF trials (n > 20,000; Vaduganathan et al.) yielded consistent HRs of approximately 0.86 for all-cause and CV mortality across the ejection-fraction spectrum, with minimal heterogeneity by diabetes status, age, sex, or eGFR [42]. EMPEROR-Preserved [4] confirmed benefit in HF with preserved ejection fraction (HFpEF) see Table 2. An updated analysis by Usman et al., published in 2024, confirmed consistency of effect across the cardiometabolic spectrum [43]. Current guidelines endorse SGLT2is as the standard of care in HFrEF, HFpEF, and chronic kidney disease (CKD), regardless of diabetes status [44].

While the cumulative clinical trial evidence places SGLT2is among the most consequential advances in HF pharmacotherapy in recent decades, contextualizing this within a specific historical comparison (for example, comparisons to the angiotensin-converting enzyme (ACE) inhibitor era) requires caution, as such comparisons are inherently dependent on methodology, era, and the baseline mortality of trial populations.

### 3.3. Intermittent Fasting: CV Biology Without the Trial Infrastructure

The absence of phase III CV outcome trials for IF reflects the non-patentability of dietary behaviors and resulting funding constraints, not biological implausibility. Evidence rests on mechanistic risk factor modification, epidemiological cohort data, and selected clinical investigations.

Mechanistically, IF consistently reduces body weight, visceral adiposity, blood pressure (3–8 mmHg), fasting insulin, homeostatic model assessment for insulin resistance (HOMA-IR), triglycerides, and high-sensitivity CRP in prospective trials [35,45,46]. Sutton et al. demonstrated improvements in insulin sensitivity, blood pressure, and oxidative stress markers with early TRE in pre-diabetic men even without weight loss, implicating chronobiological effects independent of caloric restriction [30]. Wilkinson et al. showed that 10 h TRE reduced weight, blood pressure, and atherogenic lipids in patients with metabolic syndrome [47].

The most frequently cited epidemiological observation is from Horne et al. (Utah cohort): routine 24 h monthly fasting was associated with approximately 45–50% lower odds of coronary artery disease on angiography after multivariable adjustment [48], and with lower HF hospitalization rates [49]. These findings are from a retrospective, single-center, predominantly Latter-Day Saint population and are subject to substantial confounding by health-conscious behaviors, dietary practices, and socioeconomic status not fully captured in adjustment models. They should be interpreted as hypothesis-generating rather than providing causal evidence of CV protection from fasting per se (see Table 2).

A fasting-mimicking diet program integrated into primary care reduced T2DM medication requirements in a 12-month RCT [50]. The intersection of IF and HF rehabilitation has theoretical merit in obese HFpEF, where SGLT2is reduce hospitalizations but evidence for IF’s additive role is currently speculative [51].

**Table 2:** Selected cardiovascular and metabolic outcome evidence.

Pathway	Metformin	SGLT2 Inhibitors	Intermittent Fasting
All-Cause Mortality	Moderate UKPDS 36% ↓ at 10 years [34]; meta-analysis HR 0.67 in T2DM+CAD [52]. No completed RCT in normoglycemic adults.	High EMPA-REG 32% ↓ [3]; HF meta-analysis (n > 20,000) HR 0.86 [42]. Consistent across DM/no-DM subgroups.	Very Low No RCT outcome data. Observational cohort shows lower long-term mortality in routine fasters [49], subject to substantial confounding.
CV Mortality	Moderate UKPDS ~40% ↓ in overweight subgroup [36]; meta-analysis HR 0.81 in T2DM+CAD [52].	High EMPA-REG 38% ↓ [3]; HF meta HR 0.86 [42]; EMPEROR-Preserved composite 21% ↓ [4].	Very Low Indirect via BP, weight, and inflammation reduction. No controlled outcome data.
Heart Failure	Low Safe in stable HF; observational benefit in T2DM+HF only [39]. No dedicated RCT.	High ~30% ↓ HF across trials; HFrEF (DAPA-HF [5], EMPEROR-Reduced [6]); HFpEF (EMPEROR-Preserved [4]). Guideline standard [44].	Very Low Indirect via weight/BP (especially obese HFpEF). Multimodal rehabilitation paradigm supported [51], not tested as standalone outcome intervention.
MI/Atherosclerosis	Moderate UKPDS 39% ↓ MI [36]; confirmed by systematic review. Modest lipid benefits.	Low MACE reduction primarily in established-CVD subgroup; no consistent stroke benefit; effect driven mainly by BP/weight change [53].	Very Low Reduces LDL, TG, BP, visceral fat (surrogate markers). Epidemiological signal for ↓ CAD [49]. No MI-prevention RCTs.
T2DM Prevention	Moderate–High DPP: single large, well-conducted RCT, 31% ↓ T2DM progression vs. placebo [54].	Low Not a primary indication; β-cell preservation and weight benefits observed as secondary/exploratory findings.	Moderate RCT-based evidence (synthesized in narrative review) that IF reverses pre-diabetes via weight loss and improved insulin sensitivity [55].

**Certainty of evidence (adapted GRADE framework):** High = multiple concordant RCTs and/or RCT-based meta-analyses, consistent and precise, guideline-endorsed. Moderate–High/Moderate = single well-conducted RCT, or RCT-based meta-analysis/subgroup with some imprecision or lack of replication. Low = observational evidence only (no dedicated RCT), or RCT evidence limited to surrogate/indirect endpoints, or inconsistent across trials/subgroups. Very Low = mechanistic rationale or uncontrolled epidemiological association only; no controlled clinical outcome data. Certainty ratings were assigned per cell based on the study design, consistency, and directness of the underlying evidence for that specific outcome, following a simplified GRADE approach; they should not be read as a formal, independently conducted GRADE assessment, and are intended to aid qualitative comparison across the three interventions rather than to substitute for outcome-specific systematic review. BP—blood pressure; CAD—coronary artery disease; CVD—cardiovascular disease; DM—diabetes mellitus; DPP—Diabetes Prevention Program; HF—heart failure; HFrEF—heart failure with reduced ejection fraction; HFpEF—heart failure with preserved ejection fraction; HR—hazard ratio; MACE—major adverse cardiovascular events, MI—myocardial infarction; LDL—low-density lipoprotein; RCT—randomized clinical trial; UKPDS—United Kingdom Prospective Diabetes Study; T2DM—type 2 diabetes mellitus; TG—triglyceride.

## 4. Beyond CV Disease: Cancer Chemoprevention and Gut Microbiome Remodeling

An underappreciated dimension of all three interventions is their shared capacity to attenuate cancer risk and remodel the gut microbiome. The evidence base is at varying stages of development and requires careful interpretation by study design. Colorectal cancer (CRC) is the paradigmatic shared endpoint because both metformin and SGLT2is achieve high luminal concentrations, and because IF profoundly modifies the luminal environment in which tumor genesis may be initiated.

### 4.1. Metformin: A Multi-Mechanistic Anti-Tumor Agent

#### 4.1.1. Clinical and Epidemiological Evidence

Evans et al., in 2005, first reported a 21–25% reduction in overall cancer risk in T2DM patients on metformin [56]. Noto et al. provided a meta-analytic confirmation (risk ratio (RR) 0.67 for overall cancer; RR 0.68 for CRC specifically) [57]. The landmark chemoprevention RCT by Higurashi et al. demonstrated that low-dose metformin (250 mg/day) significantly reduced colorectal adenoma recurrence in non-diabetic post-polypectomy patients at 12 months, providing level 1 evidence for direct chemopreventive activity in human colorectal mucosa [58]. Currie et al. demonstrated lower colon and pancreatic cancer incidence in diabetic patients on metformin compared with those on insulin or sulfonylureas [59]. Interpretation of observational evidence in this domain is limited by confounding, indication, healthy-user bias, and the fact that T2DM itself increases cancer risk, making comparisons between anti-diabetic agents inherently complex.

#### 4.1.2. Molecular Mechanisms

Metformin’s anti-tumor activity operates through two complementary arms: The indirect (systemic) arm functions via reduction in circulating insulin and IGF-1, a major mitogenic signal for colorectal, breast, and pancreatic cancer cells overexpressing insulin and IGF-1R receptors. The direct (cellular) arm capitalizes on high intestinal epithelial metformin concentrations (estimated 100–300-fold above plasma). Transported via organic cation transporter 1 (OCT-1) and 3 (OCT-3), metformin activates AMPK, suppresses mTORC1, forces cell-cycle arrest at G0/G1, and triggers autophagy. In vitro and animal data show selective targeting of cancer stem cells (CSCs) and inhibition of epithelial–mesenchymal transition (EMT) via vimentin downregulation and E-cadherin upregulation [13,60]. Reduction in Ki67 has been demonstrated in pre-surgical studies in endometrial and breast cancer, but these are small mechanistic studies requiring confirmation in adequately designed chemopreventive trials.

#### 4.1.3. Gut Microbiome Remodeling by Metformin

Metformin accumulates in the intestinal lumen at concentrations far exceeding plasma levels, enabling direct microbiome remodeling with potential therapeutic relevance for CRC prevention [61]. Metformin may induce several microbial shifts, with proposed downstream effects on colorectal mucosal biology (Table 3). Causal attribution of these microbial changes to cancer prevention in humans is not yet established.

### 4.2. SGLT2 Inhibitors: Metabolic Reprogrammers of the Tumor Environment

#### 4.2.1. Clinical and Epidemiological Evidence

Early concerns about SGLT2i oncogenicity (particularly bladder cancer with dapagliflozin from pooled short-term trial data) have not been confirmed by longer-term follow-up. A meta-analysis by Xu et al. found SGLT2i use to be associated with a reduction in malignancy risk compared with other anti-diabetic agents across randomized trials and observational studies [7]. A large cohort study from Asian populations (2026) reported significantly lower incidence of CRC and gastric cancer with SGLT2is, implicating SGLT1/2 transporter expression in gastrointestinal (GI) tumor biology [62]. These findings are preliminary and subject to residual confounding; they should not be presented as established cancer prevention until confirmed by prospective, adequately powered trials.

#### 4.2.2. Molecular Mechanisms

SGLT2i anti-tumor mechanisms operate through four pathways: First, obligatory glycosuria (approximately 88–90 g/day) reduces systemic glucose availability, disadvantaging cancer cells reliant on aerobic glycolysis (Warburg effect) and with reduced metabolic flexibility compared with normal cells [63]. Second, SGLT1/2 transporters are overexpressed in multiple tumor types, including pancreatic adenocarcinoma, prostate cancer, and CRC; canagliflozin and dapagliflozin (which have weak and generally not clinically relevant SGLT1 cross-reactivity at usual therapeutic concentrations) [64] may directly block sodium-coupled glucose influx in tumor cells, activating caspase cascades [65,66,67]. Third, the SGLT2i-induced rise in β-OHB acts as an endogenous histone deacetylase (HDAC) inhibitor (functionally analogous to butyrate), reprogramming tumor-cell epigenetics. Fourth, SGLT2is reduce visceral adiposity and IL-6 secretion from dysfunctional adipose tissue, attenuating the JAK/STAT3 axis implicated in CRC survival and angiogenesis. Mechanisms 2–4 are based predominantly on in vitro and animal data and require clinical validation.

#### 4.2.3. SGLT2is and the Gut Microbiome

SGLT2is modify gut microbial ecology primarily through systemic effects (glycemic and metabolic) rather than direct luminal drug concentration (Table 4) [68].

### 4.3. Intermittent Fasting: Tumor Suppression Through Metabolic Cycling

IF exerts anti-tumor effects through mechanisms that overlap with, but are temporally distinct from, pharmacological interventions. During fasting, β-OHB elevation inhibits HDAC, autophagy induction provides tumor-suppressive activity by clearing pre-neoplastic damaged cells and protein aggregates, and reduced IGF-1 withdraws a mitogenic signal from early-stage tumor cells. Animal studies consistently demonstrate delayed spontaneous tumor genesis in cancer-prone rodent strains and reduced atherosclerotic lesion development in apolipoprotein E (ApoE)-deficient mice [69]. Human epidemiological data are largely indirect (lower T2DM prevalence in routine fasters, which is itself a major CRC risk factor [48,49]). Dedicated prospective trials examining IF and cancer incidence in humans do not yet exist.

Mechanistic Synthesis: Why All Three Interventions Converge on CRC-Relevant Pathways:

- All three reduce circulating insulin/IGF-1—the primary systemic tumor-growth signal.
- All three elevate β-OHB—an endogenous HDAC inhibitor active at the luminal and systemic levels.
- All three increase butyrate-producing intestinal bacteria or butyrate-like signaling.
- All three suppress NF-κB and IL-6/STAT3 signaling in the tumor microenvironment.
- All three induce autophagy that preclinically eliminates cancer stem cell-like populations.
- Important Caveat: These parallel mechanisms do not establish clinical equivalence in cancer prevention; the strength and hierarchy of clinical evidence differs substantially across the three interventions.

## 5. Longevity and Organ Protection: Preclinical Evidence

The following preclinical findings are presented as mechanistic evidence informing translational hypotheses, rather than as established clinical claims. Extrapolation from rodent or invertebrate data to human longevity is inherently uncertain and is qualified throughout.

### 5.1. Metformin

Metformin extends lifespan by 20–40% via AMPK and microbial folate/methionine pathways—an effect abolished by AMPK knockout [70,71,72]. In the Interventions Testing Program (ITP) UM-HET3 mouse model, Martin-Montalvo et al. reported 5–6% median lifespan extension at low doses in male mice when started in mid-life [73]. More consistently, metformin improves healthspan metrics in rodents: lower inflammatory cytokines, preservation of gait speed and immune competence, attenuation of age-related left-ventricular hypertrophy, and neuroprotection in Alzheimer’s disease models [74]. Whether these findings translate to human healthspan extension is the central question of the TAME trial [2].

### 5.2. SGLT2 Inhibitors

Miller et al. reported that canagliflozin extends median lifespan by 14% in male UM-HET3 mice at clinically relevant exposures—a sex-specific effect potentially related to differential hepatic drug metabolism [75]. Organ-specific benefits documented in rodents include preserved hippocampal neurogenesis and reduced neuroinflammatory glial activation with canagliflozin [76], empagliflozin-mediated rescue of liver senescence and improved survival in naturally aged mice [77], and consistent retardation of renal aging (glomerulosclerosis, tubular senescence). Katsuumi et al. demonstrated SGLT2i-mediated senescent-cell elimination with downstream alleviation of pathological aging phenotypes [26]. Human translational studies are warranted.

### 5.3. Intermittent Fasting

IF is among the most phylogenetically conserved longevity interventions. In *C. elegans*, every-other-day feeding extends lifespan by 15–40% via RHEB-1/TORC1 modulation [78]. In rodents, alternate-day fasting (ADF) extends lifespan comparably to 30% continuous caloric restriction while preserving physical performance, cognition, and cardiac contractility [79]. IF delays spontaneous tumorigenesis in cancer-prone strains, reduces amyloid pathology in ADF models, and prevents diet-induced steatohepatitis. Non-human-primate caloric restriction trials show trends towards reduced metabolic disease and cancer, with some longevity signals, although small sample sizes and species differences limit extrapolation to human healthspan.

## 6. A Stage-Stratified Lifespan Framework

Recognizing that each intervention occupies a distinct niche in the cardiometabolic disease continuum, rational clinical deployment requires stratification by biological age, metabolic phenotype, and disease stage. The framework below integrates CV, metabolic, and oncological evidence, explicitly coding evidence strength. This framework represents expert opinion informed by the evidence reviewed and should not be applied without clinical assessment of individual risk, comorbidities, and patient preferences (Table 5).

### 6.1. Safety Profiles: Navigating the Risk Landscape

#### 6.1.1. Metformin

Gastrointestinal intolerance (nausea, diarrhea, flatulence) affects approximately 20% of patients on immediate-release formulations; extended-release formulations substantially reduce this rate. Lactic acidosis is exceedingly rare (<10 cases per 100,000 patient-years) and virtually confined to patients with eGFR < 30 mL/min/1.73 m^2^, hemodynamic compromise, or hepatic failure, due to a pharmacokinetic (accumulation) rather than pharmacodynamic mechanism [18]. Vitamin B12 deficiency occurs in 6–10% of long-term users via ileal intrinsic factor competition; periodic monitoring and supplementation are advisable [81]. Metformin should be held during acute illness with hemodynamic compromise and withheld 48 h before iodinated contrast administration. In highly active, exercise-training individuals without metabolic disease, the risk–benefit balance of off-label longevity use warrants explicit discussion [19,20].

#### 6.1.2. SGLT2 Inhibitors

Genitourinary candidal infections affect 5–10% of women and are less common in men (balanitis); these are generally manageable with hygiene measures and antifungals [84]. Euglycemic DKA is the most clinically dangerous complication and can occur without diabetes mellitus, particularly in HF patients with reduced pancreatic reserve. Risk is compounded by surgical fasting, severe illness, very-low-carbohydrate diets, and concurrent prolonged IF. Sick-day rules must be systematically communicated to all patients: discontinue the drug if unable to eat, vomiting, febrile, or fasting voluntarily for >24 h [27]. Volume-depletion-mediated adverse effects (orthostatic hypotension, early eGFR decline) require monitoring, especially in the elderly and those on diuretics. The CANVAS amputation signal was concentrated in patients with pre-existing peripheral artery disease (PAD) or active foot ulcers [40].

#### 6.1.3. Intermittent Fasting

Initial adaptation (1–2 weeks) involves hunger, mild headache, lightheadedness, and fatigue—a ‘fasting flu’, generally resolving as ketone oxidation capacity develops. Adequate water and electrolyte intake is essential. Alcohol during fasting represents an underappreciated danger: alcohol inhibits hepatic gluconeogenesis and ketone production, creating a window of hypoglycemia risk in individuals on insulin or sulfonylureas. Environmental heat stress (complete fluid restriction during Ramadan in tropical climates) adds genuine risk of dehydration and acute kidney injury. Medication adjustment is mandatory in insulin-treated diabetes [80]. IF is contraindicated in pregnancy, lactation, children and adolescents, individuals with a history of eating disorder, and those with active malnutrition. Patients should be advised that IF is a medical intervention requiring monitoring, not a self-directed lifestyle experiment when comorbidities or polypharmacy are present.

### 6.2. Future Studies

Pressing research priorities include the following: completion of the TAME trial; prospective, adequately powered IF outcome trials in cardiometabolic disease; head-to-head or combination trials across all three interventions; and human translational studies validating the senolytic and anti-tumor preclinical observations.

Future studies are needed to establish the following aspects:

- IF produces CV mortality benefit (no RCT outcome data exist);
- SGLT2is prevent cancer in humans (emerging signal, not established);
- Combination of all three produces additive clinical benefit (mechanistic inference only, no clinical trial data);
- Metformin extends human lifespan (TAME trial ongoing, hypothesis not proven).

### 6.3. Study Limitations

This narrative review has several limitations. Although metformin, SGLT2is, and IF appear to converge on several nutrient-sensing pathways (AMPK activation, mTOR inhibition, improved mitochondrial function, enhanced autophagy, and reduced inflammation), the current evidence remains largely indirect. Much of the mechanistic evidence is derived from preclinical models, while definitive human studies confirming these pathways as mediators of improved healthspan and longevity remain limited.

The evidence supporting anti-aging, cancer-preventive, and lifespan-extending effects is considerably stronger in experimental models than in healthy human populations. Most clinical data for metformin and SGLT2is originate from patients with T2DM or high CV risk, making extrapolation to metabolically healthy individuals uncertain. Similarly, the long-term adherence, safety, and efficacy of IF vary across populations and fasting protocols.

Generalizability is further constrained by the demographic composition of the underlying trials. Women have been consistently underrepresented in the SGLT2i cardiovascular outcome trials underpinning current HF guidelines, comprising well under half of most study cohorts; meta-analyses of these trials report a smaller—though still significant—relative risk reduction in major adverse cardiac events among women compared with men: an effect more plausibly attributable to reduced statistical power than to a true biological difference [85]. Metformin’s cardiovascular and cancer-preventive evidence base is drawn almost entirely from patients with T2DM, and only the ongoing TAME trial is prospectively testing outcomes in metabolically healthy, non-diabetic older adults (aged 65–79); no equivalent prospective outcome trial exists for younger or middle-aged non-diabetic populations. For IF, sex-specific data remain particularly sparse: available evidence suggests women may show greater sensitivity of reproductive and neuroendocrine axes to energetic stress than men, yet dedicated, adequately powered outcome trials stratified by sex or age are lacking [86]. Future trials across all three interventions should prioritize balanced enrollment of women, older adults, and non-diabetic individuals to allow reliable subgroup inference rather than post hoc extrapolation.

A further limitation concerns causal inference itself. Most of the epidemiological evidence linking metformin, SGLT2is, or IF to reduced cancer incidence or extended healthspan is derived from observational cohorts, which remain vulnerable to confounding by indication and to time-related biases—most notably immortal time bias, whereby drug-exposed patients are implicitly guaranteed a minimum survival time before exposure classification, artificially inflating the apparent protective effect [87]. Mechanistic and preclinical (largely rodent) data, while informative for hypothesis generation, frequently fail to translate into clinical benefit: species differences in metabolic rate, lifespan, and dosing exposure, along with the artificial uniformity of laboratory models, limit their external validity, and clinical trials targeting aging biology face additional challenges in selecting relevant populations, endpoints, and biomarkers that are not encountered in disease-specific trials [88]. Consequently, the mechanistic convergence described in this review should be interpreted as hypothesis-generating rather than as evidence of a causal, generalizable geroprotective effect.

Finally, the field is rapidly evolving. Ongoing randomized controlled trials evaluating geroscience interventions, including metformin and other longevity-targeted therapies, may substantially modify current understanding of their effects on healthspan, CV outcomes, and cancer prevention. Therefore, the conclusions presented here should be interpreted as a synthesis of the current evidence, rather than as definitive clinical recommendations.

These limitations underscore the need for well-designed, long-term randomized trials directly comparing pharmacological and lifestyle interventions, incorporating standardized biomarkers of biological aging, CV health, cancer incidence, and patient-centered outcomes.

## 7. Conclusions

Metformin, SGLT2is, and IF, at their molecular core, rely on a common biology of cellular energy scarcity, stress resilience, and regenerative signaling that constitutes an evolutionarily conserved framework for longevity. Each achieves AMPK activation, mTORC1 suppression, autophagic renewal, mitochondrial quality control, inflammasome silencing, and—as this review has sought to document with explicit attention to evidence quality—anti-tumor activity through shared metabolic pathways.

Their differences are as instructive as their similarities. SGLT2is have achieved consistent mortality benefit in HF across the ejection-fraction spectrum, with emerging oncological signals that position them as systemic metabolic reprogrammers. Metformin’s unique value resides in its direct intestinal cancer-preventive activity, supported by level 1 RCT evidence in post-polypectomy patients, along with its unmatched body of epidemiological evidence linking metabolic disease modification to reduced CV and oncological risk over decades. IF, requiring no prescription, with low pharmacological risk in healthy, normal-weight, overweight, or obese individuals, and with no cost, remains the most evolutionarily validated and culturally embedded of the three strategies.

Rather than a prescriptive algorithm, the framework proposed here is our interpretive synthesis of the evidence reviewed above: IF emerges as a form of primordial prevention accessible before disease onset, metformin as a metabolic disease modifier once dysregulation is established, and SGLT2is as organ-protective therapy once cardiorenal disease is manifest. This staging reflects the relative maturity and directness of the evidence for each intervention at each disease stage—strongest and most direct for SGLT2is in established cardiorenal disease, intermediate and RCT-supported for metformin in metabolic disease modification, and largely mechanistic-to-epidemiological for IF in primary prevention—rather than a claim that one intervention is causally superior to another within a given stage. We interpret two points as important for safe clinical translation of this framework: (1) the SGLT2i–prolonged fasting combination carries a genuine risk of euglycemic DKA and should be managed with sick-day rules; and (2) metformin should not be deployed in highly exercise-trained individuals without explicit discussion of its potential to attenuate training-induced healthspan benefits. As with the framework itself, these points reflect our reading of the current evidence and clinical judgement, not established consensus guidelines.

## Figures and Tables

**Table 1:** Mechanistic effects of metformin, SGLT2i, and IF on six aging-related pathways.

Pathway	Metformin	SGLT2 Inhibitors	Intermittent Fasting	Evidence Level
AMPK Activation	Potent—complex I inhibition raises AMP:ATP; parallel lysosomal PEN2/ATP6AP1 mechanism at low doses [12].	Sustained—obligatory glycosuria + β-OHB elevation raise AMP:ATP in cardiac and renal tissue [22].	Phasic—peaks 12–16 h post-meal with hepatic glycogen depletion; drives ketogenesis [8].	Metformin: in vitro/animal (lysosomal PEN2 mechanism established in cell culture and murine models; direct human tissue confirmation pending) [12]. SGLT2is: preclinical (mechanistic review synthesizing animal/in vitro data) [22]. IF: mixed—animal mechanistic data plus human observational/RCT support [8].
mTORC1 Inhibition	Via TSC1/2 phosphorylation (AMPK-dependent) + Rag GTPase pathway (AMPK-independent) [14].	β-OHB blunts mTORC1; AMPK-mediated Raptor phosphorylation in cardiac and renal tissue [22].	Nutrient and amino acid deprivation; most potent known physiological suppressor [31].	Metformin: in vitro/animal (cell-based and murine xenograft data) [14]. SGLT2is: preclinical (mechanistic review) [22]. IF: mixed—preclinical plus human observational data [31].
Autophagy	AMPK → ULK1-pS555; relief of mTOR-mediated ULK1 inhibition [13].	Autophagic flux is restored in the obese/diabetic kidneys and heart [23].	Strongest trigger; autophagosome formation peaks at 24–48 h fast [32].	Metformin: in vitro/animal (mechanistic review) [13]. SGLT2is: animal (obese/diabetic mouse kidney and heart) [23]. IF: preclinical (animal); human autophagic flux data remain indirect/biomarker-based [32].
Insulin/IGF-1	↓ Hepatic gluconeogenesis; improved peripheral insulin sensitivity; ↓ IGF-1 [33].	Glucosuria → ↓ plasma insulin; raised glucagon–insulin ratio [34].	Eliminates insulin/IGF-1 during fast phase; re-feeding avoids chronic hyperinsulinemia [29].	Metformin: human clinical (epidemiological and mechanistic review with RCT-supported glycemic/IGF-1 data) [33]. SGLT2is: human clinical (physiological/pharmacodynamic studies in patients) [34]. IF: human clinical (review of RCT data) [29].
ROS Mitochondria	↓ ROS via mild complex I inhibition + Nrf2→SOD2/catalase induction; preserves NO bioavailability [15].	↓ROS; restores Mfn1/2↑/Drp1↓ balance; normalizes ΔΨm and ATP synthesis [24].	↓ ROS; mitophagy removes dysfunctional mitochondria; biogenesis on re-feeding [8].	Metformin: preclinical (in vitro/animal mechanistic review) [15]. SGLT2is: preclinical (in vitro/animal mechanistic review) [24]. IF: mixed—animal and human data [8].
Inflammation and SASP	NF-κB↓; NLRP3↓; IL-1β/IL-6↓; AMPK/FOXO limits SASP secretion [16,17].	NLRP3 silenced by β-OHB; glucose deprivation undermines senescent cell metabolic dependency [25,26].	CRP↓, TNF↓; visceral fat and adipose macrophage inflammation reduced [35].	Metformin: in vitro (human cells) + animal [16,17]. SGLT2is: animal/in vitro [25,26]. IF: human clinical (meta-analysis of RCTs)—strongest evidence tier in this table [35].

Legend: AMP—adenosine monophosphate; AMPK—adenosine monophosphate kinase; ATP—adenosine triphosphate; ATP6AP1—ATPase H^+^-transporting accessory protein 1; CRP—c reactive protein; Drp1—dynamin-related protein 1; IGF-1—Insulin-like Growth Factor 1; FOXO—forkhead box O; IL-1β—interleukin-1 beta; mTORC1—rapamycin complex 1; Mfn—mitofusin; NF-κB—nuclear factor kappa-light-chain-enhancer of activated B cells; NO—nitric oxide; Nrf2—nuclear factor erythroid 2-related factor 2; β-OHB—β-hydroxybutyrate; ROS—reactive oxygen species; PEN2—presenilin enhancer 2; ULK1—Unc-51-like autophagy-activating kinase 1; SASP—senescence-associated secretory phenotype; SOD2—superoxide dismutase 2; TNF—tumor necrosis factor; TSC1/2—tuberous sclerosis; ΔΨm—mitochondrial membrane potential.

**Table 3:** Key microbial shifts induced by metformin, and their proposed downstream effects on colorectal mucosal biology.

Microbial Change	Physiological Effect	Impact on CRC Risk
↑ *Akkermansia muciniphila*	Goblet cell stimulation → MUC2/MUC5 mucus thickening	Prevents bacterial translocation and LPS-driven genotoxic inflammation; reinforces epithelial barrier
↑ Butyrate-producing species (*Bifidobacterium*, *F. prausnitzii*)	↑ SCFA production—butyrate, propionate	Butyrate inhibits HDAC in colonocytes → cell-cycle arrest and apoptosis in transformed cells; preserves tight junctions
↓ Secondary bile acid metabolism	Reduces conversion of primary to secondary bile acids (DCA)	DCA is a known genotoxic carcinogen; its reduction lowers oncogenic pressure in colorectal epithelium
Restoration of tight junction proteins (ZO-1)	Reduces intestinal permeability	Prevents LPS translocation → TLR4/NF-κB activation; reduces IL-6/TNF-α sustaining early tumor microenvironment

Legend: DCA = deoxycholic acid; HDAC = histone deacetylase; IL-6 = interleukin-6; LPS = Lipopolysaccharide; MUC2/MUC5 = genes that encode gel-forming mucins; NF-κB = nuclear factor kappa-light-chain-enhancer of activated B cells; SCFA = short-chain fatty acid; ZO-1 = Occludin; TLR4 = Toll-like receptor 4; TNF-α = tumor necrosis factor-alpha.

**Table 4:** Proposed microbial shifts generated by SGLT2is.

Microbial Change	Mechanism	Intestinal/Cancer Impact
↑ Bacteroidetes–Firmicutes ratio	Altered systemic carbohydrate availability modifies intestinal epithelial secretions	↓ Visceral adiposity, ↓ metabolic syndrome—major CRC risk driver
↑ *Roseburia, F. prausnitzii*	Improved intestinal perfusion via ↓ AGEs in microvasculature	↑ Butyrate → HDAC inhibition; preserves colonocyte tight junctions
↓ Enterobacteriaceae (including colibactin-producers)	↓ Systemic low-grade inflammation	↓ Systemic low-grade inflammation ↓ Genotoxic DNA strand breaks in colonocytes; ↓ LPS-driven NF-κB

Legend: AGE = advanced glycation end products; CRC = colorectal cancer; DNA = deoxyribonucleic acid; HDAC = histone deacetylase; LPS = Lipopolysaccharide; NF-κB = nuclear factor kappa-light-chain-enhancer of activated B cells.

**Table 5:** Stage-stratified clinical framework for integrated deployment.

Life Stage/Phenotype	Intermittent Fasting	Metformin	SGLT2is	Key Caution
Healthy adult, no metabolic risk (20–50 yr)	✓ First-line. TRE 16:8 or 5:2. Circadian alignment; data supportive [8].	✗ No RCT benefit. May attenuate exercise adaptations; inadvisable in highly trained individuals [19,20].	✗ No approved indication. Genitourinary infection and euglycemic DKA risk without benefit. Avoid SGLT2i + prolonged fasting (>24 h).	Ensure adequate protein intake with IF.
Overweight/ obese, pre-metabolic syndrome	✓✓ Highly effective: visceral fat reduction, insulin sensitivity, BP [35,45,46].	◑ Consider if pre-diabetes is present. DPP data support benefit [54].	◑ Exploratory. Consider if metabolic syndrome + elevated CV risk confirmed. Drug adjustment essential (SU, insulin) when adding IF [80].	Exclude active eating disorders.
T2DM ± established CVD	◑ Adjunct to medication; requires glucose monitoring. Avoid strict protocols on insulin	✓✓ First-line standard of care. Robust CV outcome data across multiple trials [36,52].	✓✓ Guideline-recommended [44]. Reduces MACE, HHF, and CV mortality. Do NOT combine SGLT2is + prolonged fasting.	Sick-day rules essential [27].
HF (HFrEF or HFpEF)	◑ Potentially helpful in obese HFpEF; contraindicated in cachectic HF; monitor closely.	◑ Safe in stable HF; avoid eGFR < 30 [37]. No dedicated HF mortality RCT.	✓✓✓ Guideline standard; ~30% ↓ HHF [4,5,6,42]. Use regardless of DM status.	Hold SGLT2is perioperatively, during illness, and with any fasting >24 h [27].
Frail elderly, advanced CKD (>75 yr, eGFR < 30)	✗ Avoid strict fasting; cautious modified TRE with adequate protein only.	✗ Contraindicated at eGFR < 30. Vitamin B12 monitoring if higher eGFR [18,81].	✓ Renal protection trial data to eGFR ~25–30. Benefit in >80 yr HF patients [82,83].	Volume depletion and orthostasis: monitor BP and eGFR closely. SGLT2i-induced eDKA risk.

Evidence coding: ✓✓✓ = Multiple RCTs, guideline standard; ✓✓ = ≥1 major RCT; ✓ = Single RCT or consistent evidence; ◑ = Limited or indirect evidence; ✗ = No evidence or contraindicate. Legend: BP = blood pressure; CV = cardiovascular; DKA = diabetic ketoacidosis; DM = diabetes mellitus; DPP = Diabetes Prevention Program; eGFR = estimated glomerular filtration rate; FHpEF = heart failure with preserved ejection fraction; FHrEF = heart failure with reduced ejection fraction; HF = heart failure; HHF = hospitalization for heart failure; IF = intermittent fasting; MACE = major adverse cardiovascular events; RCT = randomized controlled trial; TRE = time-restricted eating; SGLT2is = sodium–glucose cotransporter-2 inhibitors.

## Data Availability

No new data were created or analyzed in this study. Data sharing is not applicable to this article.

## Abbreviations

The following abbreviations are used in this manuscript:

AMPK	Adenosine monophosphate kinase
ATCVD	Atherosclerotic cardiovascular disease
BP	Blood pressure
CAD	Coronary artery disease
CSCs	Cancer stem cells
CKD	Chronic kidney disease
CV	Cardiovascular
CVD	Cardiovascular disease
DM	Diabetes mellitus
DPP	Diabetes Prevention Program
EMT	Epithelial–mesenchymal transition
G0/G1	Cell-cycle arrest
GLP-1	Glucagon-like peptide-1
GLP-1RA	Glucagon-like peptide-1 receptor agonists
HF	Heart failure
HFrEF	Heart failure with reduced ejection fraction
HFpEF	Heart failure with preserved ejection fraction
HR	Hazard ratio
IF	Intermittent fasting
LDL	Low-density lipoprotein
MACE	Major adverse cardiovascular events
MI	Myocardial infarction
mTORC1	Rapamycin complex 1
OCT-1	Organic cation transporter 1
RCT	Randomized clinical trial
RR	Risk ratio
SGLT2i	Sodium–glucose cotransporter-2 inhibitor
T2DM	Type 2 diabetes mellitus
